# An Updated Review on the Complex Association of Cardiovascular Disease (CVD) and Depression

**DOI:** 10.2174/011573403X337113250212093810

**Published:** 2025-03-20

**Authors:** Namra Aziz, Tanya Tripathi, Anurag Rawat, Uttam Prasad Panigrahy, Darshan Jogi Chandrashekhar, Mukesh Chandra Sharma, Pranay Wal

**Affiliations:** 1 Department of Pharmacy, PSIT-Pranveer Singh Institute of Technology (Pharmacy), NH-19, Kanpur, 209305, Uttar Pradesh, India;; 2 Department of Cardiology, Himalayan Institute of Medical Science, Dehradun, India;; 3 Faculty of Pharmaceutical Science, Assam Down Town University, Sankar Madhab Path, Gandhi Nagar, Panikhaiti, Guwahati, Assam, 781026, India;; 4 Department of Pharmacy Practice Yenepoya Pharmacy College & Research Centre, Yenepoya Deemed to be University, Ayush Campus, Naringana, Mangaluru, 575018, India;; 5 School of Pharmacy, Devi Ahilya Vishwavidalaya, Indore, 452001, India

**Keywords:** Depression, cardiovascular disease, biomarkers, therapeutic approaches, heart disease, myocardial infarction

## Abstract

**Introduction:**

The presence of both cardiovascular disease (CVD) and depression is common, and their complex connection poses difficulties in therapy and affects patient outcomes. Thus, this study aims to examine the complex correlation between depression and cardiovascular disease (CVD), with a specific focus on potential biomarkers and innovative therapeutic approaches.

**Methods:**

Publications were considered between 2015-2024 from standard databases like Google Scholar, PubMed-Medline, and Scopus using standard keywords, “Depression”, “Cardiovascular Disease”, “Biomarkers”, and “Therapeutic Approaches”. Recent studies have discovered several potential biomarkers linked to depression and cardiovascular disease (CVD), including neuroendocrine factors, inflammatory markers, and signs of oxidative stress. Therapeutic approaches for depression and cardiovascular disease have emerged, with a focus on tackling their connections from multiple dimensions.

**Results and Discussion:**

Emerging research suggests that depression has an impact on both the prognosis and risk of CVD. Conversely, depression can be caused by CVD, which triggers a series of events that lead to higher rates of illness and death.

**Conclusion:**

A comprehensive understanding of the fundamental pathophysiological pathways is essential for the identification of biomarkers that can serve as diagnostic tools or therapy targets. Among these interventions, exercise and dietary adjustments have shown promising impacts on cardiovascular health and results, as well as mental health. Ultimately, the selection of diagnostic techniques and treatments hinges on comprehending the complex interplay between depression and CVD. Researchers are developing novel therapeutic techniques to enhance the cardiovascular and mental health outcomes of individuals with both depression and CVD.

## 1 INTRODUCTION

Depression and cardiovascular disease (CVD) continue to be the world's top causes of death, making them serious global health issues. Patients with CVD frequently experience depression, especially following significant CVD events. According to studies, significant depressive disorders affect 17% to 27% of patients with coronary artery disease (CAD) who are admitted to the hospital. Moreover, depression symptoms occur in 30% of patients with coronary heart disease (CHD) and 35% of individuals with myocardial infarction but nonobstructive coronary arteries. On the other hand, the main risk factor for CVD is clinical depression. Regardless of age or gender, depression gradually raises mortality, re-hospitalization rates, and morbidity in patients with CVD. Routine depression screening is advised for all CVD patients to improve prognosis because of the reciprocal association between depression and CVD [1-3].

Thus, we aim to examine the complex correlation between depression and cardiovascular disease (CVD), with a specific focus on potential biomarkers and novel therapies to target them for treatment to increase the quality of life.

## 2 LITERATURE SEARCH

Publications considered between 2015-2024 from standard databases like Google Scholar, PubMed-Medline, and Scopus using standard keywords, “Depression”, “Cardiovascular Disease”, “Biomarkers”, and “Therapeutic Approaches”.

## 3 RESULTS

### 3.1 Pathophysiology of Depression and CVD

Depression, a serious mental disorder, has been linked to sickness and death caused by cardiovascular disease (CVD). Previous studies have confirmed a mutual relationship between the two factors [4-7]. This study investigates the molecular mechanisms involved in the development of depression and their connection to the pathways that cause cardiovascular disease (CVD) [8-11]. Fig. (**1**) illustrates the correlation between chronic stress in depression and the disturbance of endothelial function, platelet activation, and signaling pathways, all of which ultimately contribute to the development of cardiovascular disease.

Depression and cardiovascular disease are pathophysiological disorders that include several systems associated with inflammation, hormone regulation, heredity, behaviour, and the neurological system. The hypothalamic-pituitary-adrenal (HPA) axis has been linked to severe depression when it is activated during stressful situations. Patients with Major Depressive Disorder (MDD) have consistently been found to have higher cortisol levels. This hypercortisolemia leads to an elevated risk of developing a metabolic syndrome-like condition characterised by increased visceral fat mass, hyperlipidemia, and glucose intolerance. Studies have demonstrated that this metabolic syndrome leads to heightened sympathetic activation, as well as an elevated likelihood of developing diabetes, cardiovascular disease, and other ailments [12, 13]. Depressed individuals display heightened amounts of cortisol in their blood and saliva. The possibility of blood clot formation is subsequently increased due to alterations in the amounts of monoamines or catecholamines inside platelets. Numerous serotonin receptors are found in platelets, and activation of 5-HT2 receptors regulates both the clumping of platelets and the constriction of blood arteries in the heart [14]. Evidence has shown that platelets obtained from individuals with depression exhibit reduced densities of 5-HT transporter sites and increased densities of 5-HT2 binding. In addition, depression is associated with increased levels of P-selectin, activated glycoprotein IIb/IIIa, platelet β-thromboglobulin, and platelet factor four (PF4). Increased risk of cardiovascular disorders, such as ischemic heart disease, can result from depression-related alterations in platelet functions [15]. An unhealthy lifestyle, characterised by habits such as smoking, excessive alcohol consumption, poor dietary choices, non-adherence to prescribed medications, and lack of physical activity, is commonly linked to depression and can lead to weight gain, obesity, and adverse health consequences [16]. Depression-induced stress elevates glucocorticoid and glucose levels *via* gluconeogenesis, hence heightening the susceptibility to cardiovascular disease (CVD) and contributing to obesity and insulin resistance. Depression onset and subsequent cardiovascular events are associated with genetic variables. The relationship between neurotrophic brain-derived neurotrophic factors (BDNF) and steroid hormones is demonstrated by a study [17] in which the increase of glucocorticoids brought on by stress lowers BDNF levels and related brain processes. Different neurological conditions have altered the levels of BDNF in the central nervous system (CNS), including Parkinson's disease, Alzheimer's disease, cerebrovascular accident (stroke), and mental problems [18]. According to available data, glucocorticoids suppress the synthesis of microRNA-132, which lowers the activation of glutamate receptors that are BDNF-dependent. MicroRNA-132 plays a part in neuroplasticity-the brain's capacity to adapt and change. It might also have an impact on how depression and cardiovascular health coexist. It also demonstrates how depression and cardiovascular illness are related to one another [18]. This link may be impacted by additional putative genetic markers, such as Proprotein convertase subtilisin/kexin type 9 (PCSK-9) and catechol-O-methyltransferase (COMT) enzyme activity. Adopting strategies and treatments for depression and heart-related risk factors is essential to reducing the incidence of heart disease and deaths from heart-related issues. Long-lasting inflammation is the hallmark of chronic inflammatory disease (CID) [10]. The pro-inflammatory acute phase cytokines interleukin-1 and -6 (IL-1, IL-6), C-reactive protein (CRP), adhesive cell-surface glycoproteins (sVCAM-1), monocyte chemotactic protein-1 (MCP-1), cell adhesion molecules (leukocyte (L-), endothelial (E-, and platelet (P-) selectin), and intracellular adhesion molecule-1 (sICAM-1) can be used to track it [19]. As monocyte and T cell receptors, these indicators work in tandem with vascular cell adhesion molecule (VCAM-1). Adhesion molecules and inflammatory mediators are examples of peripheral clinical indicators that are used to detect artery wall inflammation. Atherosclerosis, myocardial infarction or stroke risk, and arterial wall inflammation are all strongly correlated with these indicators [20]. They have a high degree of short- and long-term prediction power for inflammatory chronic diseases. Pro-inflammatory macrophage cytokine excess might be confused with symptoms of Major Depressive Disorder (MDD), which often include hypothalamic-pituitary-adrenal (HPA) axis activation that is increased [21] (Table **1**).

Interleukins (IL-1, IL-6) directly promote the release of pituitary corticotropin (ACTH) and hypothalamus corticotropin-releasing factor (CRF). Pro-inflammatory cytokines generate resistance to glucocorticoid receptors and interfere with serotonergic neurotransmission. Consequently, there's a good chance that inflammation and major depressive disorder (MDD) are related [29].

### 3.2 Biomarker Depression and CVD

#### 3.2.1 Inflammatory Biomarkers

It has been established that inflammatory biomarkers, such as hs-CRP, can predict the likelihood of coronary heart disease (CHD) events. Studies have shed light on the cellular and molecular mechanisms *via* which these markers contribute to the development of atherosclerosis [30]. Although depression and hs-CRP have been linked to CHD events, little is known about how they might interact. An elevated hs-CRP level was found to have a 64% higher likelihood among individuals with a prior history of severe depression, as indicated by an examination of NHANES III data. On the other hand, hs-CRP levels and depression were found to be significantly and noticeably correlated in men [31]. One significant aspect of immunological dysregulation in depression and cardiovascular disease is the presence of inflammatory biomarkers. In both disease states, the interleukin (IL) cytokine group-specifically, IL-1β, IL-6, IL-8, IL-10, and IL-12 is one of the most researched indicators of inflammation. IL-6, which is produced at the site of inflammation in response to tissue damage and serves as a pro- and anti-inflammatory cytokine that regulates cellular activities, is of particular interest [32]. Pro-inflammatory cytokines are produced and recruited by tumor necrosis factor alpha (TNF-α), which also plays a significant role as an early modulator of the inflammatory response [33]. Both TNF-α and IL-1β have the ability to increase the production of the IL-6 gene within the endothelium.

Through its actions on the HPA axis, IL-6 is also in charge of promoting the synthesis of cortisol and the expression of the acute-phase protein C-reactive protein (CRP) [34]. Another immune-response mediator that is mostly produced by activated T cells in response to inflammation is the pro-inflammatory cytokine interferon gamma (IFN-γ) [35]. C-reactive protein (CRP). Five identical subunits are noncovalently linked to form the disc-shaped pentamer of CRP seen in human serum. The protein has two unique sides: an effector side and a binding side that interacts in a calcium-dependent manner with ligands. The 206 amino acid subunits of the pentameric isoform, which have a molecular weight of 23 kDa, need two calcium ions [36]. Calcium exhibits stability under physiological conditions until it forms a bond with one of its specific ligands. When calcium is present in the extracellular milieu, and its pH is within the typical range, these circumstances are referred to as phospholipids, especially lysophospholipids, which are present on the outer layer of injured or dying cells, are of special interest to the organism. It goes through monomerization after attaching to one of its ligands [37]. It appears that a number of behavioural and psychological factors, especially depression, raise the risk of Acute Coronary Syndrome episodes regardless of the presence of conventional risk factors [38]. According to a meta-analysis of 11 cohort studies, depression, whether measured by official psychiatric evaluation or self-reported symptoms, strongly predicts risk for initial CHD occurrences, even in the absence of known CHD risk factors [39]. In a similar vein, increased inflammatory biomarkers-specifically, hs-CRP-have been identified as risk factors for incident CHD events [40], and studies have detailed the cellular and molecular processes *via* which these biomarkers promote the formation of atherosclerosis [41]. Even though both depression and hs-CRP are linked to CHD occurrences, nothing is known about how they might be related.

##### 3.2.1.1 Interlukin-6 (IL-6)

Gene found on chromosome 7p15-p21 and consists of five exons and four introns. A 212 amino acid precursor protein that is split into a mature 184 amino acid portion and a 28 amino acid signal sequence is produced by the IL6 gene. IL-6 belongs to a family of proteins that use glycoprotein 130 (gp130), commonly referred to as CD130, to send messages [42-44]. The complex that binds the ligand IL-6 is the result of the interaction between the gp130 signal-transducing component and the IL-6 receptor (IL-6R). Both the pro-inflammatory trans-signaling pathway and the anti-inflammatory classical pathway can be involved in IL-6 signaling. IL-6 attaches itself to the classical pathway's IL-6R, which is affixed to the cell membrane [44]. IL-6, on the other hand, binds specifically to IL-6R, a soluble membrane-free protein, in the inflammatory pathway. The Gp130 proteins bind to the membrane on both sides of the complex once it has stabilized there. The pro-inflammatory mechanism involves the trans-signaling pathway, which is utilized by multiple types of brain cells. If the soluble, non-cellular membrane-bound gp130 proteins function as antagonists by attaching to the soluble IL-6R/IL-6 complexes, signaling will be suppressed [32]. Multifunctional cytokine mature IL-6 plays a part in many biological processes, including the formation of bone marrow, the activation of osteoclasts, the proliferation and differentiation of B cells, the development of brain fever, and the synthesis of acute phase proteins in the liver. Since IL-6 functions as a cytokine and a myokine in the immune system, it affects several autoimmune diseases [32]. The impact of IL-6 on several illnesses, including diabetes, rheumatoid arthritis, atherosclerosis, prostate cancer, and encephalitis, is well-known. It increases inflammatory states in these circumstances by acting as a pro-inflammatory cytokine. Depression begins with psychological stress, which is a major factor. Both acute and chronic psychological stress fall into this category. Acute stress is defined as being under stress for a brief period of time, whereas chronic stress is further divided into episodic and persistent types [45]. The hypothalamic-pituitary-adrenal (HPA) axis and the sympathetic nervous system are both activated by psychological stress, according to emerging research. Psychological stress also plays a significant role in creating inflammation. Researchers looked at the relationship between long-term stress and IL-6 production in a community study. They found that people who cared for spouses who had dementia saw an average four-fold increase in IL-6 compared to those who were not carers [46]. There is a link between stress and IL-6, according to a cross-sectional study done in schools with 370 male teenage participants. Significantly higher amounts of IL-6 were found in the participants after stressful experiences in their adolescent lives. A sample of ten people showed statistically significant associations between blood levels of IL-6 and surgical stress, especially in cases of acute stress like hepatectomy [46]. These numbers might be connected to MDD traits. Kakeda *et al.*'s investigation examined people with first-episode MDD who had not yet received therapy, the connection between brain morphology and IL-6 levels. Patients with MDD had considerably reduced prefrontal brain thickness, which was found to be significantly correlated negatively with blood IL-6 levels. Lower left and right subicula, the whole hippocampus, GC-DG, and the subiculum were all shown to have decreased in volume in MDD patients with high serum levels of IL-6. Patients with MDD frequently struggle to stay focused and attentive, which can seriously impair their capacity to do daily tasks. There is a link between elevated IL-6 levels and poor attention, according to a study on sustained attention in MDD patients [44]. IL-6 levels have been shown in a prior study by Gimeno *et al.* to be predictive of cognitive symptoms of depression. A follow-up time of 11.8 years was averaged throughout the study. With respect to the cognitive symptoms of depression, these data suggest that inflammation occurs prior to depression. The initial depressed symptoms did not, however, predict IL-6 levels during follow-up. Sleep disturbances are common in those who have been diagnosed with Major Depressive Disorder (MDD). Major Depressive Disorder (MDD) was diagnosed in 44 patients in a recent study; it found a correlation between the plasma blood levels of Interleukin-6 (IL-6) and the incidence of sleep disruptions in MDD patients. Both pro- and anti-inflammatory effects can result from prolonged exposure to IL-6 [47]. Vascular smooth muscle cells, ischemic myocytes, endothelial cells, immune cells, and immune accessory cells, including monocytes and macrophages, are some of the cardiovascular components that synthesize it. The dual functions of IL-6 in controlling cardiac metabolism and inflammation particularly interest medical professionals. Cytokines are more prevalent in the bloodstream of heart failure patients. The degree of left ventricular failure and the degree of sympathetic and renin-angiotensin system activation were both directly correlated with the levels of IL-6. Reductions in heart functional class, ejection fraction, and prognosis are linked to elevated levels of IL-6 [48]. Multiple research projects have demonstrated this association. According to a recent study, there is a connection between the onset of heart failure and the increased expression of IL-6 in the heart. A trustworthy indicator of the decline is IL-6. It has not been investigated what happens when hemodynamic pressure overloads abruptly or over an extended period when pro-inflammatory cytokine genes are activated. Extended hemodynamic stress causes a transient elevation in the expression of pro-inflammatory cytokines and cytokine receptor genes [49]. Even when there were no changes made to the loading conditions, pro-inflammatory cytokine genes were still downregulated. The generation of pro-inflammatory cytokines in the heart is somewhat regulated by both load-dependent and load-independent pathways. It is uncertain if the severity of the underlying cardiac malfunction alone is the cause of the increase in circulating cytokines. Notwithstanding, research conducted in labs and on animals suggests that proinflammatory cytokines could potentially impair the cardiac muscles' capacity to contract [50].

##### 3.2.1.2 Tumor Necrosis Factor-alpha (TNF-α)

TNF-α research has connected inflammation, depression, and TNF-α. The authors claim that tracking intracellular cytokines like IL-1 can help with future clinical cardiovascular disease (CVD) risk assessment. Immune activation and the concurrent rise in TNF-α levels after acute coronary syndrome (ACS) are the usual causes of the acute illness response, which is characterised by symptoms like fever, increased slow-wave sleep, heightened sensitivity to pain, loss of appetite, loss of pleasure, mood swings, and difficulty concentrating [51].

**Fig. (2):**
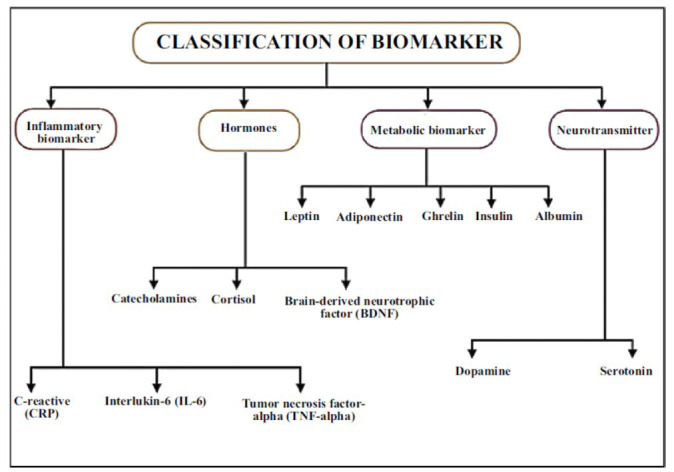
Classification of biomarkers.

Following three decades of intensive study, it is now known that this response, which is brought on by cytokines that impact parts of the CNS, is a well-preserved survival strategy that refocuses the organism's attention on energy conservation, recovery, and pathogen defense. Even though the reaction is usually flexible and reversible, in susceptible individuals, it may result in a more severe and prolonged set of behavioural and physiological alterations that are characteristic of major depression [52]. Not much data has been published thus far analyzing the association between variations in TNF-α and echocardiography parameters. TNF was inversely correlated with the E/A ratio [53] (Fig. **2**).

#### 3.2.2 Hormones

##### 3.2.2.1 Catecholamines

They impact physiological and behavioural processes that help in recovering from stress. The three catecholamines that are found naturally are dopamine (DA), noradrenaline (norepinephrine/NE), and adrenaline (epinephrine/EPI). The brain, adrenal medulla, and sympathetic nervous system all manufacture and secrete catecholamines, which have a diverse range of effects on different organs and tissues. The response to EPI and NE is mediated by a group of G protein-coupled α and/or β-adrenergic receptors (ARs); many cardiovascular drugs target these receptors. The dopamine receptors are classified into two subtypes within the G protein-coupled receptor family: D2-like receptors, which are associated with the Gi alpha subunit (Gi) (D2, D3, D4), and D1-like receptors, which are associated with the Gs alpha subunit (Gs) (D1 and D5). The primary objective of tricyclic antidepressants is to limit the neuronal uptake of NE, resulting in a reduction of depressive symptoms [54]. This supports the concept and later proof that depression may be caused by a shortage in catecholamine transmission. Nevertheless, the significance of DA was largely overlooked until recently. The availability of adrenergic and dopaminergic receptors has been linked to depression disorders, and this has, in turn, been linked to modifications in the brain's downstream pathways. Depressive illnesses have also been connected to changes in peripheral catecholamine levels [55].

Stress and depression are known to contribute to coronary artery disease (CAD) by changing the balance to one that is pro- and anti-inflammatory. They may also play a significant role in the peripheral nervous system CA-deficit, which is similar to central nervous system CA-deficit and depression CA exhaustion [56].

#### 3.2.3 Cortisol

In a state of normal bodily function and without any stress, people with good health produce approximately 10 to 20 milligrams of cortisol as part of their daily biological cycle. The circadian rhythm of the body undergoes major alterations when it is disturbed, as in the case of altered sleep-wake cycles or periods of high stress. Restoring cortisol production is a laborious process. Furthermore, the biological clock genes in the kidneys, lungs, and muscles are changed by glucocorticosteroids, which may complicate the resynchronization of the circadian clock [57]. Patients with depression have been found to have elevated levels of cortisol, the primary steroid hormone linked to the stress response system [58, 59]. Risk factors for CAD, such as high blood pressure, accelerated heart rate, increased waist-to-hip ratio, insulin resistance, diabetes, elevated total and LDL cholesterol, and decreased HDL cholesterol, have also been linked to persistent increases [60, 61].

Because heightened cortisol concentrations brought on by hyperactivity of the hypothalamic-pituitary-adrenal (HPA) axis may be linked to an increased risk of death and medical comorbidity, the association between cortisol and depression may be especially significant in patients with CAD [60, 62-63]. Ongoing stimulation of the HPA axis and high amounts of cortisol may also be important in the persistence of depressed symptoms. Cortisol, the hormone of interest, grows in hair at an average rate of one millimeter every month. In this case, the majority of the patients are indifferent and despondent.

Depression is a prevalent mental disorder worldwide. Depression is an intricate and multifaceted illness, often characterised by a low mood and decreased motivation. It may also be associated with other physical symptoms like anxiety, pain, swings in weight, and other circadian rhythm irregularities like sleep problems. Suicidal thoughts, regret, and loss of interest are common experiences for patients. Depression frequently coexists with other medical conditions, such as vitamin shortages, viral infections, cancer, hormone issues, and cardiovascular disease. Neuroendocrine issues are further associated with depression. Corticotropin-releasing hormone (CRH) is inhibited by a broken feedback loop, and anomalies in cortisol, corticotropin (ACTH), and CRH release are notable reasons in this regard [64]. The cause of the dysregulation of thyroid hormone secretion is the inability of thyrotropic hormone (TSH) to respond to the parent hormone, thyroxine (TRH), in a way that is suitable. It also has systemic effects on cardiometabolic markers. Within the circulatory system, there are two types of receptors: mineralocorticoid (MR) and glucocorticoid (GR). In addition to controlling vascular tone, glucocorticoids also affect how the body responds to damage and vascular blockage, causing tissue remodeling, cell proliferation, and inflammation. Local impacts have been suggested to have a significant impact not only on the development of atherosclerosis but also on the advancement of cardiovascular disease [65].

##### 3.2.3.1 BDNF (Brain-Derived Neurotrophic Factor)

The large number of studies conducted so far have demonstrated a causal bidirectional relationship between CVD and depression in terms of epidemiological data [66], common risk factors [67], and some specific biological mechanisms [68]. Epidemiological data on the co-occurrence of CVD and depression confirm this close bidirectional relationship and show that a patient has a higher risk of developing one of the two diseases when the other is present. Specifically, patients with depression have a 1.5-fold higher risk of developing CVD than patients without depression [68]. The prevalence of CVD in patients with major depressive disorder has been estimated to be 9.9% (95% CI: 7.4-13.3), with the cardiovascular-related mortality rate being higher than that in non-depressed patients [69]. Conversely, several studies have shown a higher prevalence of major depression in patients with CVD [70]: Major depression or an increase in depressive symptoms affects 20-45% of patients with coronary artery disease [71] and 30-40% of patients with stroke [72, 73]. According to the neurotrophic hypothesis of depression, depressive symptoms are due to a stress-induced decrease in BDNF, because of a mechanism that impairs neuroplasticity and neurogenesis and promotes cell atrophy [74], which is why decreased BDNF levels are often observed in depressed patients [75]. A more complex mechanism links BDNF and CVD: BDNF is involved in angiogenesis and promotes the survival of vascular smooth muscle cells, cardiomyocytes, endothelial cells [76], and atherosclerotic vessels. It also induces oxidative stress by activating the enzyme oxidase in coronary smooth muscle cells, which could lead to atherosclerotic plaque instability [77]. High BDNF levels play a protective role against CVD and CVD-related mortality, whereas low serum BDFN levels are considered a risk factor for future coronary events [74, 78] and are associated with an increase in the risk of future coronary events and mortality in patients with angina [76]. In line with these mechanisms of action, recent evidence has indicated that restoring normal BDNF levels through targeted therapeutic approaches reduces symptomatology in depressed patients [79] and patients with CVD [80]. Nevertheless, given that BDNF in platelets may contribute to tissue trauma or nerve damage, BDNF concentrations in serum primarily represent the activation-dependent release of BDNF from platelets, which is not as readily observed in plasma, whose coagulation-related BDNF concentrations are 200 times lower than those of serum BDNF, are extremely unstable, may be influenced by various cellular sources of plasma BDNF, have poorer repeatability and may be impacted by confounding variables like the patient's age and sex [81].

#### 3.2.4 Metabolic Marker

##### 3.2.4.1 Leptin

The polypeptide leptin is made up of 167 amino acids. Because of the obesity gene, white adipose tissue produces and releases it. The blood brain barrier (BBB) can be crossed by leptin, which then binds to a particular leptin receptor (LepRb) that is distributed throughout the brain. These regions include the cerebellum, the substantia nigra pars compacta and specific parts of the hypothalamus and thalamus. Certain cortical areas and other brain regions express this receptor at lower levels [81]. Controlling eating patterns and energy consumption is largely dependent on leptin. LepRb may, however, also affect other neurological processes, as evidenced by its presence in several brain regions. Numerous basic research investigations suggest that leptin has antidepressant qualities and may be a good target for depression treatment. Deficits in memory and cognition are caused by the absence of LepRb, and these deficiencies are correlated with modifications in the hippocampal synapses' capacity for adaptation. Mice have anxiolytic and antidepressant effects when administered leptin short-term. Additionally, research on clinical participants has demonstrated a favorable correlation between leptin mRNA and protein levels and the severity of depression. Clarifying the cellular and molecular mechanisms behind leptin's antidepressant effects and providing an overview of the information currently available are the goals of this effort [82]. We also look at the possible therapeutic applications of leptin in treating depression. The effects of leptin on the synaptic efficacy of hippocampal transmission have been found to influence both long-term depression (LTD) and long-term potentiation (LTP). Varying levels of leptin and other hormones regulated by leptin influence both long-term depression (LTD) and long-term potentiation (LTP). Research investigating the effects of leptin on hippocampal synapse function has produced contradictory findings. An intermediate dosage of leptin can be directly injected into the hippocampal region to improve long-term potentiation (LTP) and cognitive functions that rely on the hippocampal region, such as learning and memory. However, LTP is suppressed by Leptin at both higher and lower levels. Long-term potentiation (LTP) at synapses in the CA1 region of the hippocampal region may be reversed by leptin, according to research by Moult *et al.* This reversal is dependent on both the duration of exposure and the leptin concentration [83]. The promotion of long-term potentiation (LTP) by leptin aligns with its impact on cognitive processes such as learning and memory. Additionally, these effects may also contribute to the explanation of how leptin acts as an antidepressant. Glutamate disruption significantly contributes to the pathology of depression and other mood disorders associated with stress. Exposure to acute or chronic stress leads to an increase in the release of glutamate in the limbic areas of the brain. The stress-induced elevations may lead to later dysfunctional states, or the antidepressant treatments may alleviate these states. There is still disagreement and controversy over the role that leptin plays in the cardiovascular system. Leptin appears to have a part in the emergence of chronic inflammation, according to numerous research [84]. Because obesity raises levels of leptin, a hormone linked to low-grade systemic inflammation, an individual's risk of cardiovascular issues is enhanced. Moreover, elevated levels of leptin have been observed in patients with dilated cardiomyopathy, which functions as a biomarker for the progression of heart failure independent of immune responses. Four thousand eighty males between the ages of 60 and 79 who had not received a heart failure diagnosis were included in the study. For the next nine years, these men were observed [85]. Higher body mass index (BMI) and increased levels of circulating leptin were revealed to be independent risk factors for heart failure. In men without a history of coronary heart disease, higher levels of leptin were associated with an increased risk of heart failure. Body mass index (BMI) and other variables that can affect the relationship were not related to this correlation. The Framingham study, comprising 818 participants with a mean age of 79 years and 62% females, discovered a strong association between leptin levels and the development of cardiovascular disease and congestive heart failure. The relationship between leptin and congestive heart failure was no longer significant when BMI was taken into consideration [86]. The link to a higher risk of cardiovascular disease, however, was only marginally diminished. In contrast, there was no correlation found between leptin levels and the incidence of cardiovascular disease events in the Multi-Ethnic Study of Atherosclerosis, which included 1,905 randomly selected participants (average age 64.5 years, 50% women) who did not have any prior history of cardiovascular disease. This held true even after taking waist circumference, body mass index (BMI), and cardiovascular risk variables into consideration. A lot of discussion currently surrounds the application of BMI in determining cardiovascular disease risk [87]. The disagreement stems from BMI's inability to distinguish between muscle and fat mass in the body. Studies show a relationship between a higher risk of diabetes and cardiovascular diseases and visceral adiposity rather than a high body mass index (BMI). The lack of a clear correlation between leptin and the onset of heart failure is indicated by these epidemiologic findings. Leptin exposure over an extended period of time may have a detrimental impact on cardiac function [88]. Its receptors are primarily expressed in the hypothalamus and in other brain locations that regulate energy homeostasis and neuroendocrine function [89]. Very interestingly, we demonstrated that leptin is also a biomarker of stress, decreasing after exposure to a stressful environment. Leptin, also named the satiety hormone, has two main effects. The first is to stop the food intake with a negative feedback loop [90]. Leptin receptors are mainly located in the arcuate, a part of the hypothalamus that produces pro-opiomelanocortin (POMC) [91]. In earlier studies, circulating leptin levels have predicted CAD [92] and cardiovascular events in established CAD [93]. High levels of leptin have been associated with an increased risk of restenosis after percutaneous coronary intervention. Leptin levels have predicted first-ever myocardial infarction [94], and elevated leptin levels on the first morning after acute myocardial infarction have been associated with a poorer prognosis in the long term [95]. Leptin has also been an independent predictor of recurrent cardiovascular events in men with earlier acute coronary syndromes (ACSs) [96]. In addition, high levels of leptin have been associated with the incidence of stroke [97]. On the other hand, in a recent meta-analysis carried out by Chai *et al.*, there was no statistically significant connection between leptin and CAD. The authors pointed out, however, that attention should be paid to high leptin levels in men [98].

##### 3.2.4.2 Adiponectin

As an anti-inflammatory adipokine, adiponectin increases insulin sensitivity and protects against diabetes, atherosclerosis, and thrombosis. Increased synthesis of endothelial nitric oxide and a lower risk of cardiovascular disease (CVD) have been linked to elevated adiponectin levels. Conversely, plasma levels of adiponectin are lower in obese patients with cardiovascular disease (CVD) who have larger carotid artery walls (carotid intima-media thickness). Many studies have been conducted on the connection between adiponectin and depression disorders. Many studies, including a recent meta-analysis, have found that individuals with Major Depressive Disorder (MDD) had low levels of adiponectin [99]. It has also been demonstrated that antidepressant medication can successfully increase adiponectin levels. A fascinating discovery revealed an inverse correlation between adiponectin levels and the Hamilton Depression Rating Scale (HAM-D), a tool used to assess the overall duration of depression and the intensity of symptoms after admission. Nevertheless, this discovery was not validated by further investigations [100]. It is also necessary to consider other complex factors. Many variables affect adiponectin levels: body weight, metabolic syndrome, sex-specific variations in adiponectin levels, body composition and metabolic profile differences between Asians and Europeans, the type of matrix (plasma or serum) used to measure adiponectin, the occurrence of depressive events, and the various subtypes of depressive disorders. Conversely, changes in adiponectin levels may be associated with the activation of platelets and the consequent formation of blood clots. In contrast to most other adipokines, type 2 diabetes and cardiovascular disease, as well as obesity, cause adiponectin levels in the blood to drop. Obese individuals secrete less high molecular weight oligomeric adiponectin in their adipose tissue and have lower levels of adiponectin gene mRNA expression. Epidemiological studies conducted in several ethnic groups have shown that low blood levels of adiponectin, namely its HMW oligomer, can be an independent risk factor for cardiovascular disease (CVD) [101].

##### 3.2.4.3 Ghrelin

Orexigenic emotions are induced by the stomach hormone ghrelin, specifically in relation to energy balance and hunger. There is an inverse correlation between both body fat and leptin concentration. Research has demonstrated that the brain areas responsible for regulating emotions possess receptors for both ghrelin and leptin [102]. Depressed patients exhibited reduced levels of ghrelin, as determined by researchers. Twenty, however, found no noticeable difference in ghrelin levels between healthy participants and those with depression. However, multiple studies have found that persons experiencing severe depression exhibit increased levels of ghrelin in their bloodstream. Several studies investigating the impact of depression medication on ghrelin levels have shown that ECT, mirtazapine, and maprotiline all lead to a reduction in ghrelin levels [103]. The potential therapeutic applications of ghrelin in cardiovascular diseases have been hypothesized, as it has been demonstrated that the administration of the hormone to both humans and animals can reduce blood pressure, decrease cardiac afterload, and enhance cardiac output without affecting heart rate. Ghrelin strongly stimulates the pituitary gland's secretion of growth hormone (GH), energy balance, and autonomic nervous system. These effects of ghrelin may have beneficial impacts on the cardiovascular system. Moreover, it is plausible that ghrelin exerts a direct influence on the heart due to the presence of the GHS-R receptor in both the blood arteries and the cardiac ventricles [104].

##### 3.2.4.4 Insulin

A correlation has been found between depression and an increased occurrence of diabetes mellitus issues. Moreover, depression is a debilitating condition that greatly diminishes overall well-being by impairing physical health and reducing cognitive functions. When diabetes mellitus and depression coexist, there are increased risks of illness and death, poorer treatment compliance, decreased functioning, insufficient glycemic control, and higher healthcare costs [105]. A prospective study with over 4,000 individuals who had co-occurring diabetes mellitus and depression found that there was a higher risk of macrovascular problems.

Even after taking into consideration factors including the kind of treatment and prior history of problems, this risk remained. This emphasizes the gravity of diabetes mellitus in the presence of depression and emphasizes the necessity of treating both conditions concurrently. As regards cardiovascular disease (CVD), the most well-known risk factors are high blood pressure, smoking, high LDL (low-density lipoprotein) cholesterol, and type 1 or type 2 diabetes [106]. But, it is important to keep in mind that poor cardiovascular outcomes might be influenced by insulin resistance, inflammation, and hyperglycemia, or elevated blood sugar. Furthermore, there is a connection between elevated blood triglyceride and low levels of high-density lipoprotein (HDL) and the beginning of insulin resistance [107]. Moreover, research has demonstrated that approximately 30% of people diagnosed with hypertension also exhibit insulin resistance. A clear link between insulin resistance and atherosclerosis was found in 1996 by researchers conducting the Insulin Resistance Atherosclerosis Analysis (IRAS). Insulin resistance has been shown to be a major risk factor for cardiovascular disease in a subsequent prospective investigation encompassing 2938 participants [108]. Cardiovascular disease (CVD) is significantly predicted by insulin resistance, as measured by the HOMA index, according to a 2012 meta-analysis of 65 studies with 516,325 individuals. According to the study, avoiding insulin resistance could result in a 42% reduction in participant myocardial infarction rates. This finding was reached using the Archimedes model and a cohort of young adults in the 20-30 age range without diabetes. The simulated follow-up lasted sixty years. Numerous studies have presented evidence in support of the idea that there is a connection between insulin resistance and cardiovascular disease (CVD). Nonetheless, certain accounts have generated debate. According to animal models, compensatory hyperinsulinemia, a condition associated with insulin resistance, can have a significant impact on the formation of atherosclerotic plaque. This requires altering the gene expression pattern associated with the estrogen receptor. Additionally, among the metabolic and cellular processes that are affected by hyperglycemia are dyslipidemia, hypertension, endothelial dysfunction, oxidative stress, and alterations in cardiac metabolism [109].

Metformin (MET) is commonly used as a first-line therapy for patients with type 2 diabetes mellitus to minimize hepatic glucose output and improve the insulin-mediated uptake of glucose [110]. MET can reduce the adhesion of inflammatory cells to the endothelium; it also has neuroprotective, anti-inflammatory, antiapoptotic, and antioxidant properties [111, 112]. MET has been shown to enhance antidepressant efficacy and improve cognition in preclinical studies [113, 114].

Metformin also lowers risk factors for cardiovascular disease, such as blood fats [115-117], body weight and blood pressure. Compared with insulin and/or oral hypoglycemic agents (except metformin), metformin reduces the risk of all-cause mortality and the incidence of cardiovascular disease [118, 119], infection, or acidosis. Metformin can reduce the incidence of myocardial infarction (MI) in newly diagnosed obese diabetic patients [120].

##### 3.2.4.5 Albumin

The main protein in human blood plasma is called serum albumin, and it has a variety of functions. In addition, it plays a crucial role in controlling osmotic pressure and pH levels and permitting the passage of many hormones, fatty acids, and steroids. Apart from its function as a non-enzymatic antioxidant, serum albumin has also been found to play a role in the physiology of inflammation [121]. The research presented conclusive evidence of a correlation between depression and blood albumin levels in specific populations, including elderly women, HIV patients, stroke survivors, and individuals with chronic liver disease. Recent studies have established a correlation between depressed symptoms in many categories of psychiatric patients, including those with schizophrenia and individuals who have attempted suicide, and reduced levels of blood albumin [122]. Since its unintentional discovery in 1989, the concentration of SA has consistently proven to be a reliable indicator of cardiovascular disease. A thorough analysis was conducted in order to elucidate the mechanisms, validate the correlation, and expand the applicability to pertinent domains. Less SA is generally associated with unfavorable outcomes, though this relationship may not always be clear-cut. The biological functions of SA, which include antioxidant capacities, toxin binding capacity, anticoagulant effects, control of cholesterol transport, and maintenance of vascular integrity, are linked to pathophysiology. Furthermore, low nutritional intake or inflammatory diseases can also cause a decrease in SA concentration, which can impact the prognosis of cardiovascular disease [123].

Serum albumin (Alb) is the most abundant protein in plasma, representing the main determinant of plasma oncotic pressure and the main modulator of fluid distribution between the body compartments [124]. Alb is associated with coronary heart disease [125], with possible mechanisms, including responses to inflammation [126]. Moreover, the association between low Alb and increased risks of cardiovascular disease and heart failure is reported in several studies [127, 128]. In acute coronary syndrome, low Alb was associated with increased severity of coronary lesions, in-hospital mortality, heart failure, and all-cause death [129, 130]. A recent study investigated the association between low Alb concentration and adverse cardiovascular events in stable coronary heart disease, including old myocardial infarction, previous coronary artery disease, and heart failure [131]. However, the prognostic significance of low Alb level at admission in patients with newly diagnosed CAD who underwent percutaneous coronary intervention (PCI) is not well established. Therefore, we aimed to investigate the association between low Alb levels and adverse events in a retrospective cohort of patients with newly diagnosed stable CAD.

#### 3.2.5 Neurotransmitter

##### 3.2.5.1 Dopamine

There has long been a theory that the dopamine system controls circuits related to stress and depression. Acute behavioural stressors and other behaviorally relevant stimuli activate mesolimbic and mesocortical dopaminergic neurons. The prefrontal cortex's dopamine neurotransmission is significantly increased by mild stressors because the mesocortical dopamine neurons are highly responsive to acute stressful stimuli. Mesolimbic projections to the nucleus accumbens (NAc) display functional diversity, where behavioural stresses induce dopamine activity more prominently in the NAc shell compared to the NAc core or dorsal striatum [132]. Mesolimbic dopamine (DA) pathways have been identified as a major regulator of fear-related learning in studies of conditioned fear. Furthermore, the nucleus accumbens's dopamine-recipient cell sites have been directly linked to animal models of depression. It has been demonstrated that acute stress exposure raises dopamine synthesis activity in terminal areas. Different dopamine circuits exhibit this effect in different orders, with the prefrontal cortex having a larger effect than the nucleus accumbens and the nucleus accumbens having a stronger effect than the dorsal striatum. Stress is another notable risk factor for drug self-administration and relapse. The discoveries demonstrate that when individuals are exposed to acute stress, it leads to specific increases in dopamine neurotransmission and subsequent activation of signaling pathways in certain regions and cells. Studies conducted on individuals have demonstrated that the utilization of pharmaceutical treatments to inhibit or reduce dopamine (DA) has resulted in the emergence and exacerbation of depression [133]. Dopamine-β-hydroxylase, which is also found in the heart, has the ability to convert dopamine to noradrenaline [134]. Dopamine would function as a noradrenaline prodrug in this manner. No noradrenaline was produced when one constitutively deleted dopamine-β-hydroxylase was present, suggesting that dopamine-hydroxylase is the enzyme that limits the rate at which noradrenaline is created in general [135]. It would be fascinating to see how the heart's noradrenaline level would change if dopamine-β-hydroxylase were deleted specifically in the heart or even in cardiomyocytes. One may anticipate that there would be minimal noradrenaline in the heart but normal noradrenaline levels in the other tissues if the cardiac production of noradrenaline from dopamine were significant.

Moreover, dopamine is degraded by means of catecholamine-O-methyltransferase (COMT) to 3-methoxytyramine. On the other hand, selective serotonin reuptake inhibitors (SSRIs) are frequently used in conjunction with D2-like receptor antagonists and antidepressants to effectively treat major depressive disorder (MDD). Both drugs increase brain dopamine pathways. Lurasidone and aripiprazole/brexpiprazole, for instance, are novel medications that effectively reduce depressive symptoms by adjusting the dopamine receptor's activity [136].

##### 3.2.5.2 Serotonin

The fundamental causes of major depressive disorder (MDD) have been linked to the 5-hydroxytryptamine system, according to research. It has also been shown that in the brains of test animals, all antidepressants increase 5-HT signaling. On the dendritic and perinuclear spines of neurons containing 5-HT, the auto-receptor 5-HT1A is found. It might control the way that 5-HTergic neurons react to external stimuli. Long-term use of selective 5-HT reuptake inhibitors (SSRIs) was found in 2010 to be associated with functional desensitization or downregulation of 5-HT1A auto-receptors in the mid-suture dendrites [137]. This attenuated the inhibitory effects of selective serotonin reuptake inhibitors (SSRIs) on serotoninergic neurons. Stroke is a highly detrimental ailment that affects people worldwide. Even while thrombolysis and thrombectomy are beneficial treatments, it's not always simple for people to regain brain function following rescue. Post-stroke depression (PSD) is a prevalent psychiatric condition that frequently occurs in individuals who have experienced a stroke. A prospective study looked at urine and blood samples from 28 patients who had either a hyperacute ischemic stroke or a transient ischemic attack, as well as those from 29 healthy persons. The patients had higher 5-HT and 5-HT2 receptor levels than the control group [138].

Thus, it can be concluded that 5-HT may have an impact on cardiovascular and psychological diseases. Research found that between 2% and 55% of people have post-stroke depression. Though many clinical and experimental studies have been conducted on PSD, the underlying pathophysiological mechanisms are still not fully understood. However, the function of 5-HT transporters and their receptors is very important. The human heart's 5-HT4 receptor isoforms are responsible for regulating the neurotransmitter's contractile, chronotropic, and proarrhythmic effects. 5-HT receptor expressions can change because of cardiovascular illness. In ApoE-/- mice, the concurrent administration of antagonists that block 5-HT2AR and inhibitors that lower 5-HT synthesis also has a synergistic effect on reducing the formation of atherosclerotic plaques and macrophage infiltration [139]. Like hepatic steatosis, the advancement of lipid atherosclerosis is linked to the activation of intracellular 5-HT2AR, the synthesis of 5-HT, and the breakdown of 5-HT. The antagonist sarpogrelate slows down the progression of atherosclerosis in a rabbit model by decreasing the activity of 5-HT2A receptors and possibly limiting the proliferation of smooth muscle cells and macrophages by boosting the production of endothelial nitric oxide synthase (eNOS). Furthermore, studies have shown that selective serotonin reuptake inhibitor antidepressants (SSRIs) can lower the death rate and incidence of cardiovascular diseases. The impact that has been observed can be explained by the normalization of platelet abnormalities and serotonin in depressed people who get effective treatment with SSRIs. Mood regulation is probably greatly influenced by the interactions between the inhibitory metabotropic receptors 5-HT1AR and 5-HT2AR in the chemoreceptor complex [140]. This means that when 5-HT2AR prolamins are stimulated, there is a decrease in the transmission of post-conjugative 5-HT1AR prolamins in the forebrain. When the coordinated connections between the receptors in the vulnerable 5-HT1A heteroreceptor complex are broken, major depression may result. Furthermore, the suppression of the OXTR and 5-HT2AR components of the 5-HT2CR heteroreceptor complex may contribute to the development of mental illnesses such as depression, which are typified by abnormal social behaviour [141]. A study encompassing 300 individuals with coronary artery disease (CAD) found that patients with depression and cardiovascular problems had a significantly higher probability of both severe and mild adverse cardiac events [142]. Patients with Major Depressive Disorder (MDD), depression, and cardiovascular illness had increased serotonin receptor densities. However, bigger sample sizes would be necessary in future research to thoroughly investigate this connection. 5-hydroxytryptamine absorption is lowered when the S allele is present in the polymorphism area of the 5-hydroxytryptamine transporter (SERTs) gene, as this suppresses the activity of the gene. Several studies have shown that patients diagnosed with acute myocardial infarction (AMI) who carry the S allele in this specific gene region are at an increased risk of experiencing repeated episodes of heart attacks. Part of the increased risk in patients with acute myocardial infarction (AMI) is due to the presence of depressive symptoms [143].

The effects of serotonin *via* 5-HT_4_-receptors lead to positive inotropic and chronotropic effects, as well as arrhythmias, in the human heart. In addition, 5-HT_4_ receptors may play a role in sepsis, ischemia, and reperfusion. These presumptive effects of 5-HT_4_ receptors are the focus of the present review. We also discuss the formation and inactivation of serotonin in the body, namely, in the heart. We identify cardiovascular diseases where serotonin might play a causative or additional role. We address the mechanisms that 5-HT_4_ receptors can use for cardiac signal transduction and their possible roles in cardiac diseases [144].

### 3.3 Novel Therapies

#### 3.3.1 Neural Stem Cells (NSCs)

The number of studies on neural stem cells (NSCs) that have been published has significantly increased in recent years. This research has demonstrated that contrary to common thought, the adult brain still harbours multipotent NSCs, despite its often inactive and inflexible nature. NSCs are associated with tissue stem cells because of their distinct and well-known attributes, which include the ability to remain undifferentiated without a specific phenotype in certain conditions, the capability to self-renew through division and multiplication, and the capacity to differentiate into various progenies during neurogenesis, such as neurons, oligodendroglia, and astroglia. The hippocampus, subventricular zone, and neural structures are neurogenic areas in the adult mammalian brain that contain specialized competent cells [145]. These cells possess the capacity to spontaneously create neurons and respond to the creation of local signals by generating neurons. To stimulate the growth of new neurons from stem cells or progenitors, the process of neurogenesis (NG) is believed to rely on a specific set of signaling signals that need to be provided to neurogenic cells in a precise and coordinated manner by their surrounding environment. Aside from the widely recognized modulators, it is believed that damage alone is enough to trigger neurogenesis. BDNF expression additionally stimulates the formation of new neurons. Non-stem cells (NSCs) have the ability to significantly improve or restore the quality and function of brain tissue [146]. They are frequently extracted from adult brain tissues, including post-mortem brain tissue. These cells have the potential to be used in the treatment of central nervous system disorders. Neural stem cells (NSCs) are generated, genetically altered, or replicated in a controlled setting in the laboratory with the goal of altering the cell lineages of the central nervous system (CNS). Knowing how adult neurogenesis works It is predicted that activated cells will release growth factors, neuromodulators, enzymes, transmitters, tissue hormones, and antibodies into the surrounding tissue milieu, so triggering the intended tissue responses. In damaged neuronal and glial networks, these newly empowered cells and their progeny can function as helpful “healing agents” and efficient enhancers. These characteristics have greatly aided in the development of treatments for trauma and circulation problems, including stroke, ischemia, and neurodegenerative disease. Thus, it is not surprising that there is a great deal of discussion on NSCs' potential in the field of mental health treatment. Certain cellular and anatomical relationships, together with mostly unknown genetic variations, are probably present in many mental illnesses [147]. The hippocampal neuronal population is known to decline during depressive episodes. This implies that although an increase in neurogenesis can be beneficial, a lack of neurogenesis may be the underlying cause of depressive symptoms. Alleviate symptoms and regulate the antidepressant effect. However, the presence of conflicting data regarding the mechanism by which neurogenesis reduces depression has required much inquiry. Originally established before the complete validation of this mutual concept. The neural somatic cell progeny develops into fully formed neurons of the central nervous system when adult hippocampal neurogenesis is stimulated. Subsequently, these central nervous system neurons acquire the necessary morphological and functional traits to either substitute dying brain cells or assimilate into pre-existing neural networks. This review specifically examines the types of stem cells that have been utilized in recent studies on heart regeneration, among the various accessible options. These comprise the following: The blastocyst, which develops three to five days after sperm fertilizes an egg cell, is the source of embryonic stem cells (ESCs). They are the source of all the cell types found in the mature organism, with the exception of the placenta and umbilical cord [148]. When it comes to the level of specialization, adult stem cells-also referred to as somatic or tissue-specific stem cells-are more advanced than embryonic stem cells (ESCs). In living things, the primary role of adult stem cells is to preserve and rebuild the tissue in which they are found. Multipotent stromal cells from bone marrow can be isolated to produce mesenchymal stem cells (MSCs). These stem cells can differentiate into mesodermal lineage cells that comprise bone, cartilage, muscle, and fat. They do not develop into blood cells and are multipotent as well [148]. Through a process of genetic reprogramming, any type of tissue-typically skin or blood-can be used to create induced pluripotent stem cells or iPSCs. They can develop into any kind of adult cell due to their pluripotency. Because umbilical cord blood stem cells may produce all types of blood cells in the human body, they are taken out of the cord as soon as the baby is delivered. The field of stem cell research and the development of hiPSC-CMs have shown tremendous promise recently because of these cells' capacity to grow in culture for prolonged periods of time, their similarity to native human cardiomyocytes in terms of ion channels and signaling pathways, and their accessibility in large quantities [149]. Furthermore, Wu *et al.*'s research team has shown that by successfully regenerating damaged cardiac tissue, human induced pluripotent stem cells (hiPSCs) derived from sick people have the potential to provide novel therapeutics for ischemic heart disease. Apart from their well-established ability to regenerate tissue *via* the release of growth factors and cytokines, stem cells may also have an impact on the course of treatment. Trophic mediators, which are produced by stem cells, have multiple functions that improve the heart. Among these are reducing inflammation, limiting tissue damage, promoting the growth of new blood vessels, avoiding the formation of scar tissue, and liberating stem cells from the host tissue. Thymosin-β4, Wnt5a, Angiopoietin 1 and 2 (Ang-1 and Ang-2), Erythropoietin (EPO), Macrophage Inflammatory Proteins (MIP-1), Vascular Endothelial Growth Factor (VEGF), Hepatocyte Growth Factor (HGF), Insulin-like Growth Factor 1 (IGF-1), and Stromal cell Derived Factor 1 (SDF-1) are significant cardioprotective factors secreted by stem cells. Doses of FGF-2 prevent ischemia-induced cardiac arrhythmias, apoptosis, and cell death. Additionally, it promotes the production of the anti-apoptotic protein Bcl-2. Furthermore, HGF, bFGF, Ang-1, Ang-2, and VEGF released by BMMSCs promote an increase in vascular density and blood flow in the ischemic heart. Circulating progenitor cells are encouraged to be mobilized to wounded locations by SDF-1, IGF-1, and HGF, which hastens the process of healing and regeneration [150]. Between the ages of 24 and 27, stem cells suppress fibrosis by releasing matrix metalloproteinases, collagens, transforming growth factor beta (TGF-β), and tissue-derived inhibitors. These components are found in the extracellular matrix. Thus, employing the right mediator could enhance the effects of cell therapy. Regenerative heart research has made use of a variety of stem cell types, including myoblasts, endogenous cardiac stem cells, embryonic cells, umbilical cord-derived MSCs, and bone marrow-derived cells. The development of hiPSCs has resulted in significant advancement in the domains of regenerative and precision medicine. HiPSCs have the potential to be used therapeutically because, in contrast to ESCs, they are patient-specific and do not trigger an immune response. Moreover, hiPSCs can be reprogrammed using easily accessible tissue sources such as the skin, fat, or blood of the donor. Given that these cells have the potential to differentiate into adult heart muscle cells, they hold great promise for cardiac regeneration therapy. Moreover, using it could help avoid common ethical and legal controversies surrounding the use of embryonic stem cells (ESCs) [151].

#### 3.3.2 Cytochrome Genes

The Clinical Pharmacogenetics Implementation Consortium (CPIC) and the Dutch Pharmacogenetic Working Group (DPWG) have synthesized clinical recommendations that provide strong scientific evidence for the use of candidate genes implicated in antidepressant metabolism (pharmacokinetics) in clinical practice. These genes encode the cytochrome P450 (CYP450) enzymes CYP2D6 and CYP2C19, which are involved in the metabolism of antidepressants. Due to common functional genetic differences in the population, these genes' enzymatic activity varies significantly [152]. Ultrarapid metabolizers (UMs), extensive metabolizers (EMs), intermediate metabolizers (IMs), and poor metabolizers (PMs) are the four primary categories into which the variants can be categorized. It was feasible to investigate these metabolic groups' effects on drug metabolism because they were connected to pharmacokinetic characteristics, such as drug and metabolite plasma concentrations, for several antidepressants. There is little data regarding the relationship between metabolizing groups and clinical results (response/side effects) apart from tricyclic antidepressants (TCAs), escitalopram, and venlafaxine. Furthermore, it is yet unclear how plasma levels and clinical outcomes are related. For selective serotonin reuptake inhibitors (SSRIs), the concentration-response curve is anticipated to be relatively consistent, with significant variations mostly occurring at very low and high plasma concentrations [153]. This suggests that patients with markedly diminished or increased enzymatic activity (known as poor metabolizers and ultra-rapid metabolizers, respectively) may exhibit noteworthy alterations in clinical results. However, due to the rarity of these groups in the general population, there is little evidence of such effects in existing literature. Recent findings indicate that individuals with CYP2C19 poor metabolizer phenotype experience more improvement in symptoms and have a larger chance of symptom remission when treated with (es) citalopram. However, they also face an elevated risk of experiencing side effects, such as gastrointestinal, neurological, and sexual issues. These findings corroborate the suggestions put out by clinical guidelines, affirming that individuals with poor CYP2C19 metabolizer phenotypes should undergo heightened clinical surveillance and that the initial dosage should be decreased to 50% of the usual dose [154]. Nevertheless, they also proposed that individuals classified as CYP2C19 poor metabolizers should not be disregarded when considering treatment with (es) citalopram. Similar principles are applicable to suggestions for alternative antidepressants, namely Tricyclic Antidepressants (TCAs) and Selective Serotonin Reuptake Inhibitors (SSRIs). When there is a medical reason to prescribe a prescription that is predominantly broken down by a faulty or overactive enzyme, it should be avoided in individuals with poor or ultra-rapid metabolism. If necessary, the dosage should be adjusted, and careful monitoring should be conducted [155]. This document presents a summary of a report on the suggestions derived from the metabolizing activity of CYP2D6 and CYP2C19. CPIC and/or DPWG have provided pharmacogenetic biomarkers for a total of thirteen antidepressants. Guidelines currently lack definitive data on the specific populations that would benefit the most from pharmacogenetic testing, thus not providing any specific recommendations on when or to whom the testing should be advised. Patients who have not shown a positive response to or have had difficulty tolerating at least one previous treatment should undergo pharmacogenetic testing, as recommended by the latest evidence. It is unlikely that variations in CYP450 genes other than CYP1A2, CYP3A4/A5, and CYP2B6 will have a substantial clinical effect on drug metabolism. This is because environmental factors, such as nutrition, smoking, and concurrent medications, have a greater influence on their level of activity than genetic factors. Therefore, it is imperative that pharmacogenetic testing does not include polymorphisms in these genes [156].

#### 3.3.3 Genetics in Depression CVD

The patient's cardiovascular outcome and the onset of depression are influenced by genetic factors. Research points to a potential connection between steroid hormones and the neurotrophic factor BDNF. Elevations in glucocorticoids brought on by stress disrupt neurons that are associated with brain-derived neurotrophic factor (BDNF). Many neurological conditions, such as mental health problems, Parkinson's disease, Alzheimer's disease, and cerebrovascular accidents (CVAs), are associated with variations in BDNF levels within the central nervous system (CNS). The neuroplasticity of microRNA-132 implies that it may have a role in both the coexistence of depression and cardiovascular health. The relationship that depends on depression and cardiovascular disease (CVD) [157]. Type 2 diabetes is regarded as an independent cardiovascular risk factor, and patients with depression are twice as likely to develop it. Furthermore, there's a substantial correlation between depression and a 60% rise in type 2 diabetes cases. Whether or not diabetes is present, anti-hyperglycemic medications such as insulin, metformin, glyburide, pioglitazone, and others have been shown in multiple trials to reduce depressed symptoms [158]. The mechanisms by which these anti-hyperglycemic medications produce their antidepressant effects are decreased blood glucose, reduced oxidative stress and inflammation in the brain, and regulation of the HPA axis. Insulin resistance is among the numerous cardiovascular risk factors that have been linked to PCSK9, a mediator of LDL cholesterol [159]. An investigation was conducted into the relationship between depression and cardiovascular outcomes, obesity, insulin resistance, and PCSK9 levels. The Beck Depression Inventory (BDI) revealed that the study's results indicated that the obese participants had higher than average levels of PCSK9 [160]. Increased levels of PCSK9 in obese, depressed people may indicate an increased risk of cardiovascular morbidity and assist in determining which people would benefit most from targeted intervention. This is especially important because diabetes risk has been raised in those with depression. These five noteworthy research works clarify the relationship between depression and cardiovascular disease (CVD) [161].

#### 3.3.4 Physical Therapies

Physical therapists should recognize that various attributes of major depression, such as diminished interest, motivation, and energy, as well as widespread fatigue, low self-esteem and self-assurance, aversion to movement, and psychosomatic ailments, can impede engagement in physical exercise. The risk level of individuals with concurrent physical diseases must be determined, as must their physical fitness and perceived exertion during activity. To develop exercise regimens that work for these patients, physical therapists should identify individuals who are at high risk before starting treatment, such as those who have a history of diabetes or cardiovascular diseases [162-168]. These patients must obtain medical approval before beginning any physical activity [169].

Exercise regimens are usually heterogeneous, consisting of a range of formats, levels of intensity, lengths of time, and frequency. Aerobic, non-aerobic, and mind-body exercises are the three types of exercise. Exercises like walking, cycling (including ergometric, stationary, and recumbent bicycles), swimming, jogging, cross-training, jumping rope, using “transport” machines, step aerobics, cardio-kickboxing, resistance exercises with TheraBand, dumbbells, Swiss Balls, stretching exercises, free weight training, and traditional Chinese exercises like Taijiquan and Qigong, yoga, and Pilates are just a few examples of the many different types of exercises that are used in clinical research. Most of the trials supported aerobic exercise as the preferred option. A range of activities was provided for the participants to choose from to improve their adherence [170]. Furthermore, studies have shown that mind-body, aerobics, and weight training are effective ways to help people with their depression symptoms. In a research, Nassasia *et al.* used motivational interviewing (MI) as an extra therapeutic intervention in conjunction with a structured multimodal exercise plan for patients with major depressive disorder (MDD) who were between the ages of 15 and 25. Following therapy, the intervention group demonstrated improved somatic health and behavioural activation in addition to a significant improvement in the cognitive and affective subscales of the revised Beck Depression Inventory II (BDI-II). It has also been confirmed by numerous studies on older adults (65 years of age and older) with depression (152) that physical activity reduces depressive symptoms [171]. Exercise reduces cardiovascular risk factors, improves psychological health, and raises quality of life overall, in addition to easing depressive symptoms linked to diabetes and other chronic conditions. For individuals with clinical heart failure, exercise has definite benefits for depressive symptoms; these benefits irrespective of test quality, age, length of intervention, or exercise environment are important. Additionally, exercise has a particular therapeutic effect on depression symptoms as well as overall functioning in people who have heart transplants, multiple sclerosis, hemodialysis, arthritis, Alzheimer's disease, Parkinson's disease, schizophrenia, and other rheumatic conditions [172]. When a medicine is contraindicated and the body is unable to accept it, exercise therapy can be beneficial [173]. Of all the different types of aerobic activities, walking is particularly efficient in burning fat and enhancing cardiopulmonary function. Additionally, it carries a low risk of injury due to its minimal stress on the joints. Therefore, it is frequently advised as a potent workout for individuals who are obese. Walking on a Nordic track is one activity. The entire upper body, including the arms, shoulders, chest, and back, is worked out during Nordic walking, which also works on the lower body. In addition, compared to ordinary walking, it uses between 30 and 70% more energy. To our knowledge, no research has, however, really investigated walking as a kind of physical activity in this setting. In addition, earlier studies have primarily demonstrated the beneficial effects of walking and active transportation on improving certain cardiovascular risk factors in people, such as diabetes, body mass index (BMI), and hypertension [174, 175]. These activities have also been connected to a decline in the incidence of cardiovascular disease outcomes, including death, stroke, and acute coronary heart disease. No studies have investigated how often people have increasing levels of cardiovascular risk and illness walk. To better understand the cardiovascular risk levels of the population, this study set out to collect data on the frequency of walking among adult Americans. In adults with varying degrees of cardiovascular risk and illness, the study sought to evaluate the total prevalence of walking, including diverse forms of walking. Further analysis of the association between the degree of cardiovascular risk and disease and people's participation in any kind of exercise was another goal of the study. Walking for transit and walking for pleasure have different characteristics that either facilitate or impede them, according to earlier research. For this reason, we performed independent analyses for these two scenarios. Designing future interventions can benefit greatly from an understanding of the particular walking styles that are more or less prevalent in particular communities [176]. The ongoing COVID-19 pandemic is increasing the frequency of mental health issues globally, especially in those who have already been infected, healthcare workers, people in quarantine, and people with pre-existing chronic illnesses. A substantial prevalence of psychological morbidity was observed among patients with COVID-19. It was found that more than half of the population experienced psychological problems, including psychological discomfort (34%), poor sleep quality (40%), and stress (34%). Indian research conducted during the COVID-19 shutdown revealed that 74% of individuals experienced stress, while 40% exhibited indications of anxiety or depression [162]. By 2030, unipolar depression is projected to become the second leading contributor to the worldwide burden of disease. The effectiveness of yoga in treating mental health issues stems from its ability to address stress, inflammation, and autonomic dysfunction. Our primary objective was to create a reliable yoga regimen specifically designed for those suffering from mild depression. Yoga is a widely used supplementary therapy in the United States. It is especially beneficial for depression because it can be customized to suit one's daily mood by incorporating practices that improve physical, mental, and spiritual well-being. Additionally, yoga is freely accessible and may be practiced independently [163]. The deliberate and controlled breathing techniques and meditative/relaxation exercises of yoga are intended to promote a state of tranquility, overall wellness, increased ability to handle stress, and enhanced mental concentration. These effects may help reduce symptoms of depression, anxiety, stress, and excessive thinking. Yoga employs mild physical postures to improve strength, flexibility, and balance, providing practitioners of this ancient practice with a feeling of mastery over their bodies. The physical motions of yoga, which is a focused and low-impact activity, may have antidepressant and anxiolytic effects. Modern lifestyle pressures have been found to be a major contributory cause of many diseases, including CVD. An American study found that practicing mindfulness-based stress reduction (MBSR), which includes yoga, can lower inner-city primary care physicians' average number of visits [164]. This implies that yoga might be beneficial for general health, especially for those who are under a lot of mental stress, especially for heart health. Yoga has been shown in numerous trials to significantly improve risk factors for cardiovascular disease (CVD), including blood pressure, lipid profiles, body weight, smoking, psychological stress, and type 2 diabetes. Although the effects of yoga on hypertension differ, transcendental meditation (TM) was found to have an impact on blood pressure in nine carefully planned randomized controlled trials, according to a recent meta-analysis. Comparing the practice of Transcendental Meditation (TM) to the control group, there was a reduction in both the diastolic and systolic blood pressure of 3.2 mm Hg (confidence interval 1.3-5.6) and 4.7 mm Hg (confidence interval 1.9-7.4) [165]. Even if yoga and meditation may only result in a slight drop in blood pressure, they can nevertheless have a significant effect on lowering the risk of cardiovascular disease (CVD). Research indicates that a mere 3 mm Hg reduction in systolic blood pressure among the general population can potentially reduce the risk of stroke by 8% and coronary heart disease (CHD) by 5%. It is thought that a reduction in sympathetic activity and the restoration of baroreceptor sensitivity brought about by yoga practice may be the cause of the drop in blood pressure. Transcendental Meditation techniques may be assessed in clinical practice as alternative treatments to lower blood pressure (BP) and minimize the risk of cardiovascular disease (CVD), according to a recently published scientific statement from the American Heart Association (AHA). The American Heart Association (AHA) states that studies have indicated that TM may be able to reduce heart attacks, strokes, and heart attack-related deaths in individuals with cardiovascular disease [166]. The major end point, which was a composite of all-cause mortality, myocardial infarction, and stroke over a period of 5.4 years, showed a 48% risk reduction in a recent randomized controlled study of TM and health education in Blacks. Regular yoga and meditation practice significantly reduce early atherosclerosis as determined by carotid intimal medial thickness, according to two randomized trials. In comparison to the usual care control group, three randomized trials using coronary angiography in advanced cardiac heart disease have shown that yoga and meditation combined with a low-fat vegetarian diet induced a slowing of advancement and even a reversal of coronary obstructions. Furthermore, there was a significant decrease in the need for interventional procedures. Significant decreases were observed in LDL cholesterol, triglycerides, body weight, angina, and exercise-induced ischemia in the yoga group. Yoga can be a useful tactic for cardiac rehabilitation since it can improve general well-being, lower stress levels, and increase physical fitness. Yoga also promotes better sleep, food control, and a decrease in physiological arousal. The benefits of integrating yoga into cardiac rehabilitation have been demonstrated by a few small studies [167].

#### 3.3.5 Diet

The factors that influence dysbiosis include the consumption of fiber, fatty acids, and probiotics. Indeed, dietary experiments have shown that alterations in the microbiota may be caused by these specific food items. However, the occurrence of depression has not been evaluated simultaneously. There are significant negative associations between the consumption of cereal, seaweed, and mushroom fiber and the presence of depressive symptoms, according to a Korean study looking into the relationship between dietary fiber and depression. Even though the results were encouraging, the only factor that significantly correlated with pre-existing depression was the ingestion of seaweed fibre. When the lowest consumption quartile (quartile 1) was compared to the highest intake quartile (quartile 4), the multivariable-adjusted odds ratio was 0.45 (95% confidence interval: 0.23-0.88). Moreover, the initial diversity and number of bacteria in the gastrointestinal tract have a significant impact on changes in the microbiome. As was already established, the microbiome shows an amazing resistance to change that is yet not fully understood [177]. Thus, more research is needed before making general recommendations. The information that is now available suggests that eating a healthy diet, such as the Mediterranean diet, may reduce the risk of developing depression, despite some notable gaps in the scientific study. 2015 saw the creation of the first Dietary Guidelines for the Prevention of Depression by a group of experts in several disciplines, including nutrition, psychiatry, and epidemiology. Further benefits for obesity, diabetes, cardiovascular disease, and metabolic syndrome are provided by these dietary suggestions. They also pose little risk of injury.A substantial number of foods high in omega-3 polyunsaturated fatty acids (PUFAs) must be consumed. The amount of processed food, fast food, baked goods, and sweets should be limited. In addition, it is advisable to replace unhealthy food choices with wholesome and nutritious ones. Given the substantial evidence supporting diets' benefits for metabolic and cardiovascular diseases, such as UDD, these guidelines are critical in encouraging the use of diets as preventative interventions [178].

### 3.4 Neutraceuticals

Probiotics, vitamin D, PUFA, a mix of vitamins and minerals, and other supplements are comprised of the five groups [179].

#### 3.4.1 Polyunsaturated Fatty Acid (PUFA)

Various studies concluded that the PUFA group, which comprised both EPA and DHA in combination, was the most often used supplemental type for treating depression. Eight of the twelve clinical trials utilizing PUFA supplementation for depression showed promise in reducing the intensity of symptoms. Even when the other four characteristics did not significantly alter the severity of depression, most research concluded that PUFA was helpful as a treatment for depression. As we can see, the eight studies demonstrating the favorable effects of PUFA on depression had varying daily dosages of EPA and DHA, ranging from 0.7 g to 2 g, respectively. Moreover, usage of polyunsaturated fatty acid (PUFA) supplements ranged from once daily to nine times daily across a three- to four-month period. Most trials seem to show that supplementing with polyunsaturated fatty acid (PUFA) at a daily dose-that is, a total daily dose of 180 mg of eicosapentaenoic acid (EPA) and 120 mg of docosahexaenoic acid (DHA), given six times a day-produces positive outcomes [180]. However, larger dosages and longer research periods-up to three years-were employed in the trials that were unable to show a meaningful effect. However, we discovered a study by Namara *et al.* that claimed PUFA was helpful in treating depression. Extending the dosage from previous trials, the study administered 16.2 grammes (10.8 grammes of EPA + 5.4 grammes of DHA) divided into two tablespoons daily as a total daily dosage. The quantity was selected based on an earlier study that demonstrated that supplementation was beneficial and safe for people with attention deficit hyperactivity disorder. Furthermore, out of the eight studies, only one concentrated on pregnant mothers at different stages of pregnancy and demonstrated positive effects of PUFA supplementation on depression during pregnancy. The results of the Hsu *et al.* study, indicating that taking omega-3 fatty acid supplements, like EPA, in sufficient amounts during pregnancy is crucial because they are important for the development of the fetal brain and retina in addition to helping to lessen pregnancy-related depression, supported this conclusion. The anticipated duration of the pregnancy is also decided by them [181].

## CONCLUSION

It can be difficult to diagnose depression in patients who also have heart disease because two conditions often present similar signs and symptoms. The presence of depressive symptoms in patients with heart disease is likely the result of interaction between central and peripheral factors. These symptoms can be caused by several different factors. Psychological factors can activate stress pathways, which can result in an acceleration of inflammatory mediators. Thus, we may conclude that a comprehensive understanding of the fundamental pathophysiological pathways is essential for the identification of biomarkers that can serve as diagnostic tools or therapy targets. Thus, the study of potential biomarkers for patient profiling in personalized medicine approaches could be there, but the significance needs further investigation. Among these interventions, exercise and dietary adjustments have shown promising impacts on cardiovascular health and results, as well as mental health. Ultimately, the selection of diagnostic techniques and treatments hinges on comprehending the complex interplay between depression and cardiovascular disease (CVD). Researchers are developing novel therapeutic techniques to enhance the cardiovascular and mental health outcomes of individuals with both depression and cardiovascular disease (CVD).

## Figures and Tables

**Fig. (1):**
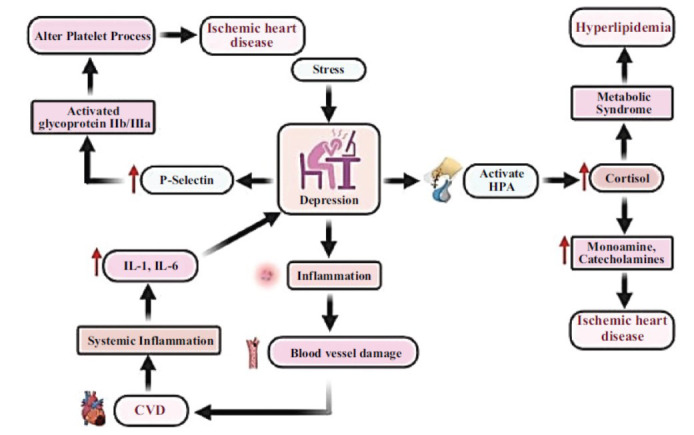
Correlation between chronic stress in depression and the disturbance of endothelial function, platelet activation, and signaling pathways, all of which ultimately contribute to the development of cardiovascular disease.

**Table 1:** Clinical data of depression related CVD.

**Country**	**Sample**	**Method**	**Results**	**Conclusion**	**References**
Korea	481 355 Male= 260 695 Female= 220 660	Cohort study ICD-10 codes F32-F33	Depression increased the risk of developing CVD by 41% for males and 48% for females.	Depressed people were at increased risk of CVD incidence.	[22]
Korea	163,024	Retrospective cohort study ICD-10 codes F32-F33	The HR between depression and ischemic heart disease was 1.38 (95% CI, 1.23 to 1.55) and depression and cerebrovascular disease was 1.46 (95%, 1.32 to 1.62).	Depression elevated elderly Koreans' risk of ischemic heart disease by 38% and cerebrovascular disease by 46%.	[23]
Sweden	292 men	Cross-sectional study	Raising triglyceride levels (*p <* 0.001), rising levels of high-sensitive C-reactive protein (hs-CRP) (*p* = 0.021), younger age (*p <* 0.001), male sex (*p <* 0.001), and depression (*p* = 0.045) are all linked to low levels of high-density lipoprotein cholesterol.	Lower levels of HDL cholesterol were shown to be independently correlated with depression, male sex, lower age, greater triglycerides, and low-grade inflammation as evaluated by hs-CRP.	[24]
Iran	7083	Cohort study	Depression and DII score are associated in women but not in males. Of the patients, 50.5% (n = 3580) had mild to severe anxiety, while 37.1% (n = 2631) had mild to severe depression.	In this Iranian group, there was a strong correlation between severe depression and DII score for women but not for men.	[25]
United States	367,703	Population-based cohort study	A 20% increase in depression risk with a family history of heart disease (95% CI 16-24%, *p <* 0.0001), but not with a genetic risk score significantly linked to coronary heart disease (CHD) risk. Rise in CHD genetic risk score led to 71% greater CHD risk and 1% higher depression risk (95% CI 0-3%; *p =* 0.11). Mendelian randomization analysis revealed that triglycerides, IL-6, and CRP may cause depression. Depression odds ratio per standard deviation increase in genetically-predicted triglycerides was 1.18 (95% CI 1.09-1.27; *p* = 2 × 10−5), 1.35 (95% CI 1.12-1.62; *p* = 0.0012), and 1.18 (95% CI 1.07-1.29; *p* = 0.0009).	Environmental factors cause depression-CHD comorbidity. IL-6, CRP, and triglycerides may also cause depression.	[26]
Hong Kong	11,651	Retrospective cohort study	1306 (11.2%) of 11,651 depressed people developed CVD. Multi-adjusted models revealed a significantly higher risk of CVD for those with depression duration of 2-5 years (HRs: 1.38 [95% CI: 1.19-1.60]) and ≥6 years (1.45 [1.25-1.68]) compared to those with depression within one year.	Long-term depression increases CVD risk significantly.	[27]
Hong Kong	500,199	Genome-wide association studies (GWAS)	Depression characteristics were linked to both Myocardial infarction (MI) (rG = 0.169; *p* = 9.03 × 10−9; broad depression: rG = 0.123; *p* = 1 × 10−4) and atrial fibrillation (AF) (rG = 0.112; *p* = 7.80 × 10−6; broad depression: rG = 0.126; *p* = 3.62 × 10−6). Genetically increasing depression odds was linked to higher risk of CAD (OR = 1.099; 95% CI 1.031-1.170; p = 0.004) and MI (OR = 1.146; 95% CI 1.070-1.228; p = 1.05 x 10−4). Adjusting for blood lipids/smoking status reduced depression-CAD/MI causation.	Positive genetic tendency to depression may cause CAD/MI.	[28]

## LIST OF ABBREVIATIONS

BDNF: Brain-Derived Neurotrophic Factor
CAD: Coronary Artery Disease
CHD: Coronary Heart Disease
CRP: C-Reactive Protein
CVD: Cardiovascular Disease
HPA: Hypothalamic-Pituitary-Adrenal
IL-1: Interleukin-1
MCP-1: Monocyte Chemotactic Protein-1
MDD: Major Depressive Disorder
PF4: Platelet Factor Four
VCAM-1: Vascular Cell Adhesion Molecule
